# Development of a Novel PET Tracer [^18^F]AlF-NOTA-C6 Targeting MMP2 for Tumor Imaging

**DOI:** 10.1371/journal.pone.0141668

**Published:** 2015-11-05

**Authors:** Qinghua Liu, Donghui Pan, Chao Cheng, Dazhi Zhang, Anyu Zhang, Lizhen Wang, Hongdie Jiang, Tao Wang, Hongrui Liu, Yuping Xu, Runlin Yang, Fei Chen, Min Yang, Changjing Zuo

**Affiliations:** 1 Department of Nuclear Medicine, Changhai Hospital, the Second Military Medical University, Shanghai, 200433, China; 2 Jiangsu Institute of Nuclear Medicine, Key Laboratory of Nuclear Medicine, Ministry of Health, Wuxi, 214063, China; 3 Department of Organic Chemistry, School of Pharmacy, Second Military Medical University, Shanghai, 200433, China; 4 Department of Pharmacology, School of Pharmacy, Fudan University, Shanghai, 201203, China; University of Manchester, UNITED KINGDOM

## Abstract

**Background and Objective:**

The overexpression of gelatinases, that is, matrix metalloproteinase MMP2 and MMP9, has been associated with tumor progression, invasion, and metastasis. To image MMP2 in tumors, we developed a novel ligand termed [^18^F]AlF-NOTA-C6, with consideration that: c(KAHWGFTLD)NH_2_ (herein, C6) is a selective gelatinase inhibitor; Cy5.5-C6 has been visualized in many *in vivo* tumor models; positron emission tomography (PET) has a higher detection sensitivity and a wider field of view than optical imaging; fluorine-18 (^18^F) is the optimal PET radioisotope, and the creation of a [^18^F]AlF-peptide complex is a simple procedure.

**Methods:**

C6 was conjugated to the bifunctional chelator NOTA (1, 4, 7-triazacyclononanetriacetic acid) for radiolabeling [^18^F]AlF conjugation. The MMP2-binding characteristics and tumor-targeting efficacy of [^18^F]AlF-NOTA-C6 were tested *in vitro* and *in vivo*.

**Results:**

The non-decay corrected yield of [^18^F]AlF-NOTA-C6 was 46.2–64.2%, and the radiochemical purity exceeded 95%. [^18^F]AlF-NOTA-C6 was favorably retained in SKOV3 and PC3 cells, determined by cell uptake. Using NOTA-C6 as a competitive ligand, the uptake of [^18^F]AlF-NOTA-C6 in SKOV3 cells decreased in a dose-dependent manner. In biodistribution and PET imaging studies, higher radioactivity concentrations were observed in tumors. Pre-injection of C6 caused a marked reduction in tumor tissue uptake. Immunohistochemistry showed MMP2 in tumor tissues.

**Conclusions:**

[^18^F]AlF-NOTA-C6 was easy to synthesize and has substantial potential as an imaging agent that targets MMP2 in tumors.

## Introduction

The matrix metalloproteinases (MMPs) are a family of more than 20 extracellular, zinc-dependent proteins that are capable of degrading multiple components of the extracellular matrix, as well as non-matrix substrates [1–6]. MMPs, particularly gelatinases A and B (MMP2 and 9), are closely associated with the metastatic potential of many neoplasias [3,4,6–11]. The importance of MMP2 and MMP9 in tumor progression, angiogenesis, and metastasis suggests that targeting them with imaging agents would be a useful strategy to noninvasively detect and characterize solid tumors.

Many investigators have imaged MMP2 in tumors using PET or single-photon emission computed tomography (SPECT) [12–15]. The most intensively studied compound in this regard is the cyclic peptide CTT (i.e., CTTHWGFTLC), discovered by Koivunen et al. [16]. This MMP2/MMP9 inhibitor has been radiolabeled and evaluated in preclinical experiments by numerous groups, including Sprague et al. [12], Hanaoka et al. [13], Kuhnast et al. [14], and Medina et al. [15]. However, selective binding to MMP2 was not demonstrated in these studies, and all modifications of this peptide exhibited poor *in vivo* stability, leading to low tumor uptake.

Because CTT is readily degraded *in vivo*, Wang et al. [17] synthesized the cyclic decapeptide c(KAHWGFTLD)NH2 or c(Lys-Ala-His-Trp-Gly-Phe-Thr-Leu-Asp)NH2 (herein, C6) by replacing the S-S bond in the cyclic peptide CTT with an amide bond between the ε-amino group of lysine (Lys) and the side chain of aspartate (Asp). The resulting peptide was more stable and exhibited a 4-fold increase in gelatinase inhibition. C6, with conjugation of a Cy5.5 near-infrared fluorescent dye molecule (Cy5.5-C6), has been visualized *in vivo* in many tumor models, such as prostate PC3, glioma U87, and inflammation-induced colon tumors [17,18]. However, this optical approach has low tissue penetrance and does not enable deep tissue localization; thus, its use in the clinic is restricted [1].

PET provides highly sensitive, noninvasive, and quantitative images of various cancers. A variety of peptides have been radiolabeled with 18-fluorine (^18^F) for use in PET imaging, because this radionuclide has low positron energy, a lack of side emissions, approximately 100% positron efficiency, and a suitable half-life. However, the process of labeling peptide with ^18^F is complex [19], and simpler ^18^F-labeling methods would be highly desirable. Recently, several one-step labeling methods have been developed via B-F [20,21], Si-F [22], and Al-F [23–25] chemistry. A simple strategy for preparing ^18^F-labeled peptides via complexation of [^18^F]-aluminum fluoride (AlF) with a NOTA-derived peptide has been introduced [19,23–25].

In the present study, C6 was selected as a mother compound, and [^18^F]AlF-NOTA-C6 was developed as a PET probe. The tumor-targeting characteristics of [^18^F]AlF-NOTA-C6 were also preliminarily investigated.

## Materials and Methods

### General information

The equipment utilized in the current study included: an HM-67 medical cyclotron (Sumitomo, Japan); an AC210S electronic balance (Sartorius, Germany); a Bond Elut (02011) C18 column (Agilent Technologies, USA); a Waters 600 high-performance liquid chromatography (HPLC) and XBridge separation column (Waters, XBridge, USA); a TR610 radioactivity detector (Perkin-Elmer, USA); an animal 50262 anesthesia machine (Stoelting, USA); an Inveon micro-PET scanner (Siemens, USA); and a 1470 Wizard gamma radioimmunoassay counter (Perkin-Elmer, USA).

**Fig 1:**
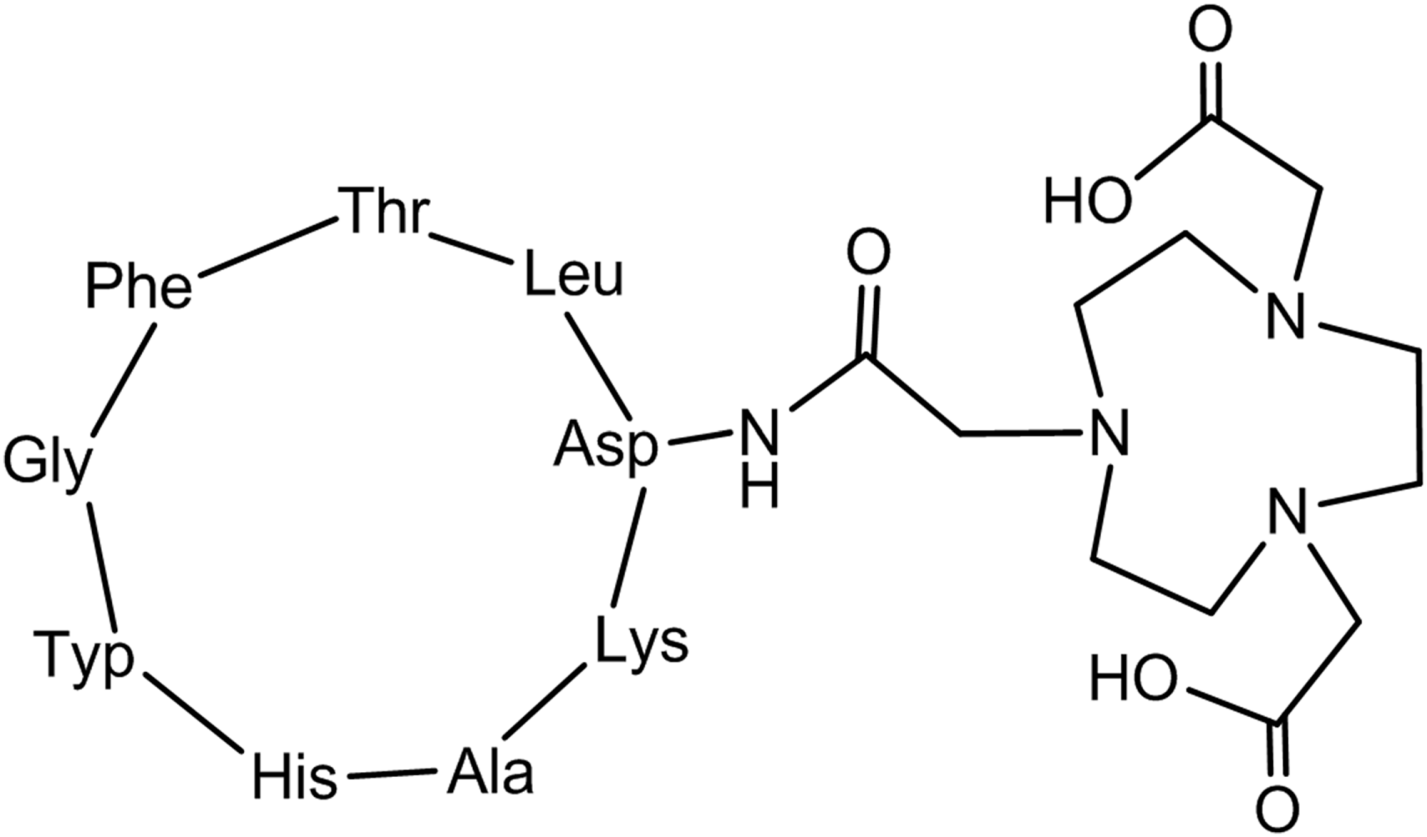
The chemical structure of NOTA-C6.

All chemicals were obtained from commercial sources and used without further purification. Acetonitrile, trifluoroacetic acid (TFA), aluminum chloride, ethanol, and glacial acetic acid were purchased from Chemical Reagent (Sinopharm). NOTA-Cyclo (Lys-Ala-His-Trp-Gly-Phe-Thr-Leu-Asp)-NH_2_, whose chemical structure was shown in Fig 1 (see S1 Fig), was purchased from Chinese Peptide (Hangzhou, China). Isoflurane was obtained from Abbott Laboratories (Shanghai, China).

### ^18^F Labeling

**Fig 2:**
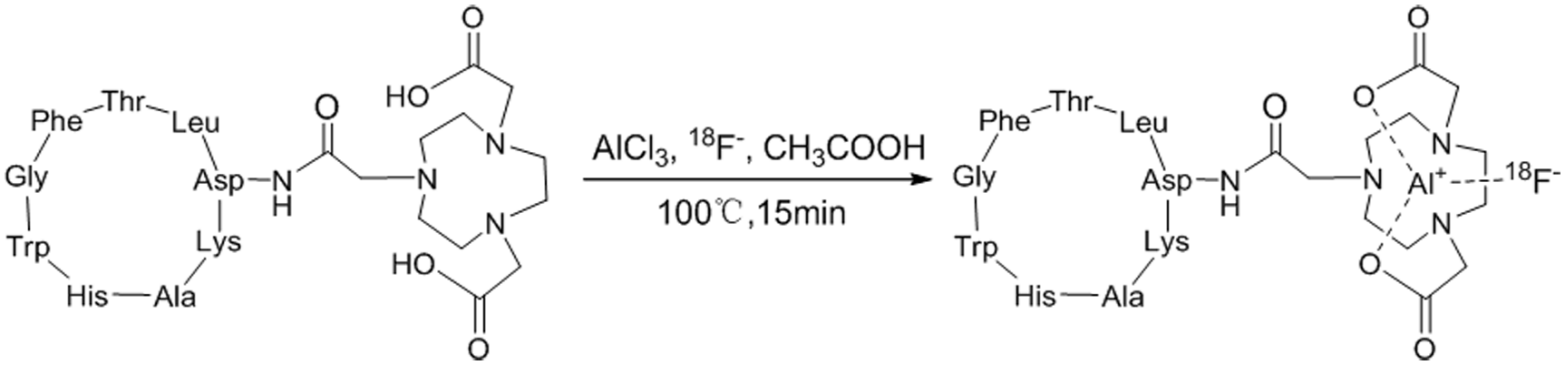
Schematic of [^18^F]AlF-NOTA-C6 radiosynthesis.

[^18^F]-fluoride was produced by a cyclotron using the ^18^O (p, n)^18^F nuclear reaction, and diluted with saline to an appropriate concentration. The method of radiolabeling NOTA-C6 with ^18^F is shown in Fig 2, which was similar to previous reports [24,25] (see S2 Fig). A 30-μL aliquot of 1 mM NOTA-C6 in 0.2 M pH 4 sodium acetate-acetonitrile buffer was added to a 1-mL vial that contained 6 μL of 2 mM AlCl_3_ in 0.2 M pH 4 sodium acetate-acetonitrile buffer, and then ^18^F^−^ (~740 MBq) in 60 μL of target water was added. The reaction mixture was heated at 100°C for 15 min. Then, the reaction mixture was cooled, diluted with 25 mL of deionized water, and loaded onto a Varian Bond Elut C18 column. The cartridge was washed again with water (25 mL), and the desired labeled peptide was eluted with 10 mM HCl in ethanol (0.3 mL). The product was reconstituted in saline and passed through a 0.22-μm Millipore filter into a sterile vial for subsequent studies.

For quality control purposes, a portion of the product was diluted and injected onto an analytical C18 HPLC column (Hydrosphere C18, 5 μm, 4.6×250 mm, UV-detection at 254 and 218 nm) to assay for radiochemical purity. A linear gradient was utilized, which started from 5% A (0.1% TFA in acetonitrile) and 95% B (0.1% TFA in water) for 2 min and ended to 65% A at 32 min at 1 mL/min. The retention time for [^18^F]AlF-NOTA-C6 was 19.3 min.

### *In vitro* stability

To examine *in vitro* stability, approximately 400 μL of [^18^F]AlF-NOTA-C6 and 2 mL of physiological saline at room temperature, or 2 mL of human serum at 37°C, were mixed and incubated for 4 h. During the incubation, 200-μL samples were collected at 2 and 4 h. The samples were subsequently analyzed by radio-HPLC.

### Cell uptake

The cell lines (human SKOV3 ovarian and PC3 prostate cancer cell lines) were obtained from the center laboratory of Changhai Hospital (Shanghai, China). Cell uptake studies were performed in accordance with the following protocol. SKOV3 cells were diluted to 5×10^6^ cells/mL in binding buffer, seeded into a 24-well plate (2.5×10^5^ cells/well), and incubated overnight. The cells were rinsed with phosphate-buffered saline (PBS), and 500 μL of fresh Dulbecco’s modified eagle’s medium (DMEM) containing 0.1% bovine serum albumin was added to the culture wells. [^18^F]AlF-NOTA-C6 (~18 kBq/well) was then added to the wells, and the incubating time was set at 5 timepoints (1, 2, 3, 4, and 5 h) in triplicate. At each given timepoint, the cell supernatants were collected in measurement tubes, washing 3 times with PBS, and the eluents of the cells were also collected in measurement tubes; the radioactive counts at this step were termed the Cellout. The cells were subsequently lysed with NaOH-sodium dodecyl sulfate (SDS; 0.5 M NaOH, 1% SDS). The cell lysates and subsequent eluents of PBS were collected via the same approach; the radioactive counts of this step were termed the Cellin. All measurements were performed with a γ-counter. Cell uptake studies were also performed on PC3 cells using the same methods.

### Competitive cell-binding assay

The binding affinities and specificities of [^18^F]AlF-NOTA-C6 were determined using NOTA-C6 as a competitive ligand, as described by other authors [26,27]. The SKOV3 cells were seeded into a 24-well plate and incubated overnight. Increasing concentrations of NOTA-C6 (0.0001–10 nM) were administered into the wells in triplicate. After 12 h of incubation, the plates were washed with PBS, and 500 μL of fresh DMEM that contained 0.1% bovine serum albumin were added to the culture wells. Then, 37 Kbq of [^18^F]AlF-NOTA-C6 was added into each well. After an additional 5h of incubation at room temperature, cellular uptake was assessed. The liquids of Cellout and Cellin were independently collected and counted with a γ-counter. The best-fit 50% inhibitory concentration (IC_50_) value was calculated by fitting the data through nonlinear regression, using GraphPad Prism (GraphPad Software, San Diego, CA, USA).

### Animal models

Animal experiments were conducted in accordance with our institutional guidelines. The Animal Care Committee of Second Military Medical University approved all experimental procedures.

Tumor-bearing mouse models were established using SKOV3 and PC3 cells. The cells were maintained in DMEM, which contained 10% fetal bovine serum (GIBCO, Grand Island, NY, USA), at 37°C in a 5% CO_2_ humidified atmosphere.

Tumor-bearing mice were prepared via a subcutaneous injection of cancer cells into the forelegs of female (SKOV3) or male (PC3) BALB/c nude mice (B&K Universal, Shanghai, China) at 6 weeks of age (body weight 18–20 g). When tumors were palpable, i.e., approximately 5 mm in diameter, the mice were used for biodistribution and PET imaging experiments.

### MicroPET imaging of tumor-bearing mice

PET imaging was performed on 4 mice bearing SKOV3 tumors using a microPET scanner; prior to the tracer injection, 2 mice were pre-blocked with unlabeled C6 at 10 mg/kg body weight, and 2 mice were not pre-blocked. Pre-blocked and non-blocked mice were then administered 3.7 MBq of [^18^F]AlF-NOTA-C6, via tail vein injection. At 30, 60, and 120 min post-injection (PI), the mice were anesthetized with 1–2% isoflurane, positioned supine, immobilized, and imaged. The scan time was 10 min. The images were reconstructed using the ordered subset expectation maximization method; no attenuation correction was used.

Imaging studies were also performed on 2 mice bearing PC3 tumors using the same methods.

### Biodistribution experiments (pre-blocked and non-blocked)

Biodistribution experiments were performed via intravenous administration of [^18^F]AlF-NOTA-C6 (0.74 MBq) to mice bearing SKOV3 tumors. At 30, 60, and 120 min PI (n = 4 mice/timepoint), the mice were euthanized and the biological samples of interest (e.g., blood, tumor, muscle, lung, spleen, kidney, liver, and small intestine) were harvested and weighed.

In the blocking experiments, the mice (n = 4) were pre-blocked with unlabeled C6 at 10 mg/kg body weight prior to the tracer injection; at 60 min PI of [^18^F]AlF-NOTA-C6, the mice were euthanized as previously described. The radioactivity values of the tissues and injection standards were subsequently measured in a γ-counter. The samples were corrected for radioactive decay to calculate the percent of injected dose (ID) to grams of tissue (%ID/g) relative to a standard that represented the injected dose.

### Histopathology and MMP2 immunohistochemistry of tumors

The histopathology of the cancer tissues was analyzed by hematoxylin and eosin (H&E) staining. To investigate the levels of MMP2 in the SKOV3 and PC3 tumors, immunohistochemical (IHC) staining was performed using an enhance labeled polymer system method (EnVision HRP DAKO, Glostrup, Denmark) in accordance with the manufacturer’s instructions. The quantitative analysis of IHC staining was performed with Image pro-plus 6.0 software (Media Cybernetics, Rockville, MD, USA).

### Statistical analyses

Data are expressed as the mean ± standard deviation. The means were compared using Student’s *t*-test (SPSS 17.0, SPSS, USA). Differences were considered statistically significant at *P* < 0.05.

## Results

### [^18^F]AlF-NOTA-C6 synthesis

**Fig 3:**
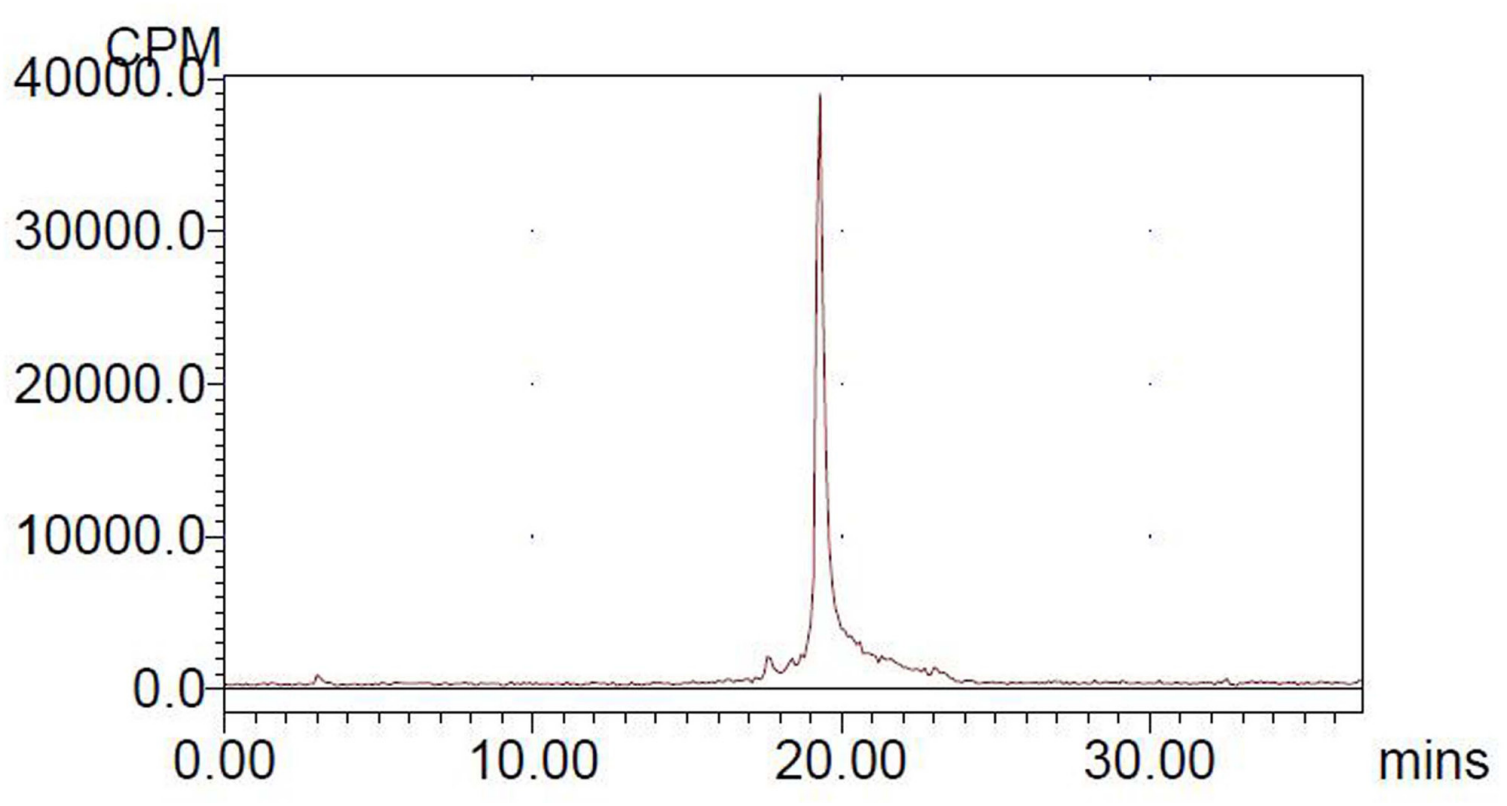
Analytical HPLC profile of [^18^F]AlF-NOTA-C6.

The labeling was performed within 30 min, with a non-decay corrected yield of 46.2–64.2%, and the specific activity was about 15.8 to 48.3 GBq/μmol. The radiochemical purity of [^18^F]AlF-NOTA-C6 exceeded 95%, as determined by HPLC. In the HPLC analysis, [^18^F]AlF-NOTA-C6 exhibited one peak at a retention time of 19.3 min, the result was given in Fig 3 (see S3 Fig).

### *In vitro* stability

**Fig 4:**
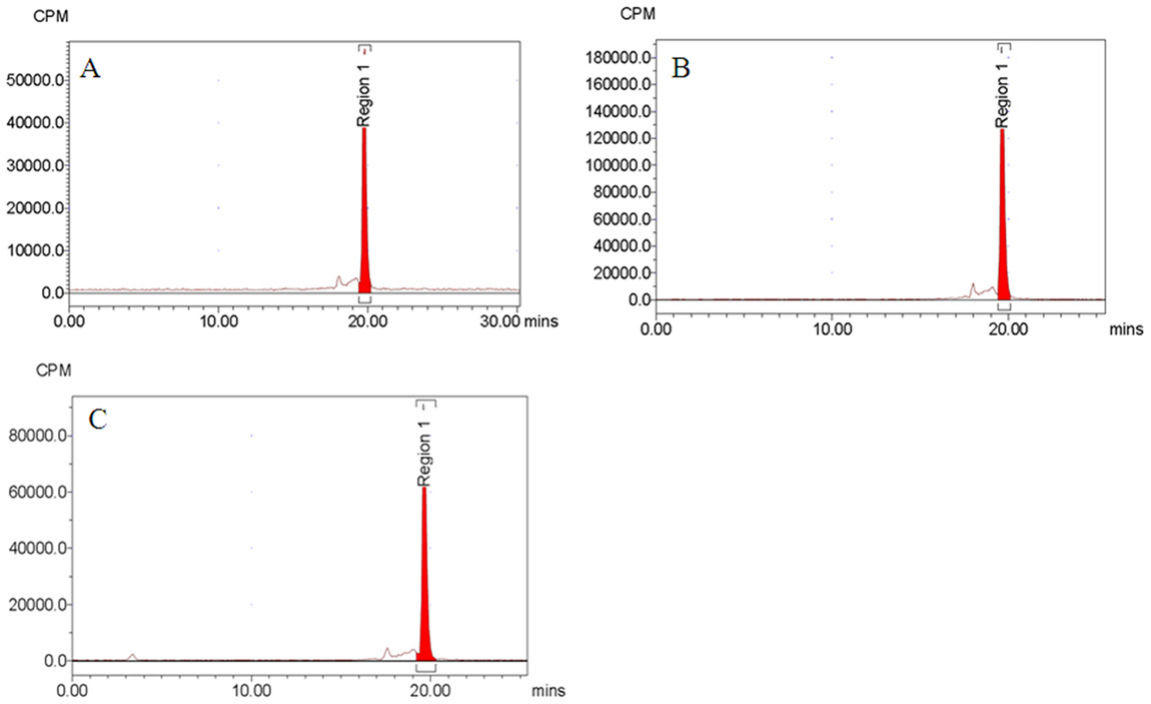
Analytical HPLC profile of [^18^F]AlF-NOTA-C6 after incubation in physiological saline at room temperature for (A) 0, (B) 2, and (C) 4 h.

**Fig 5:**
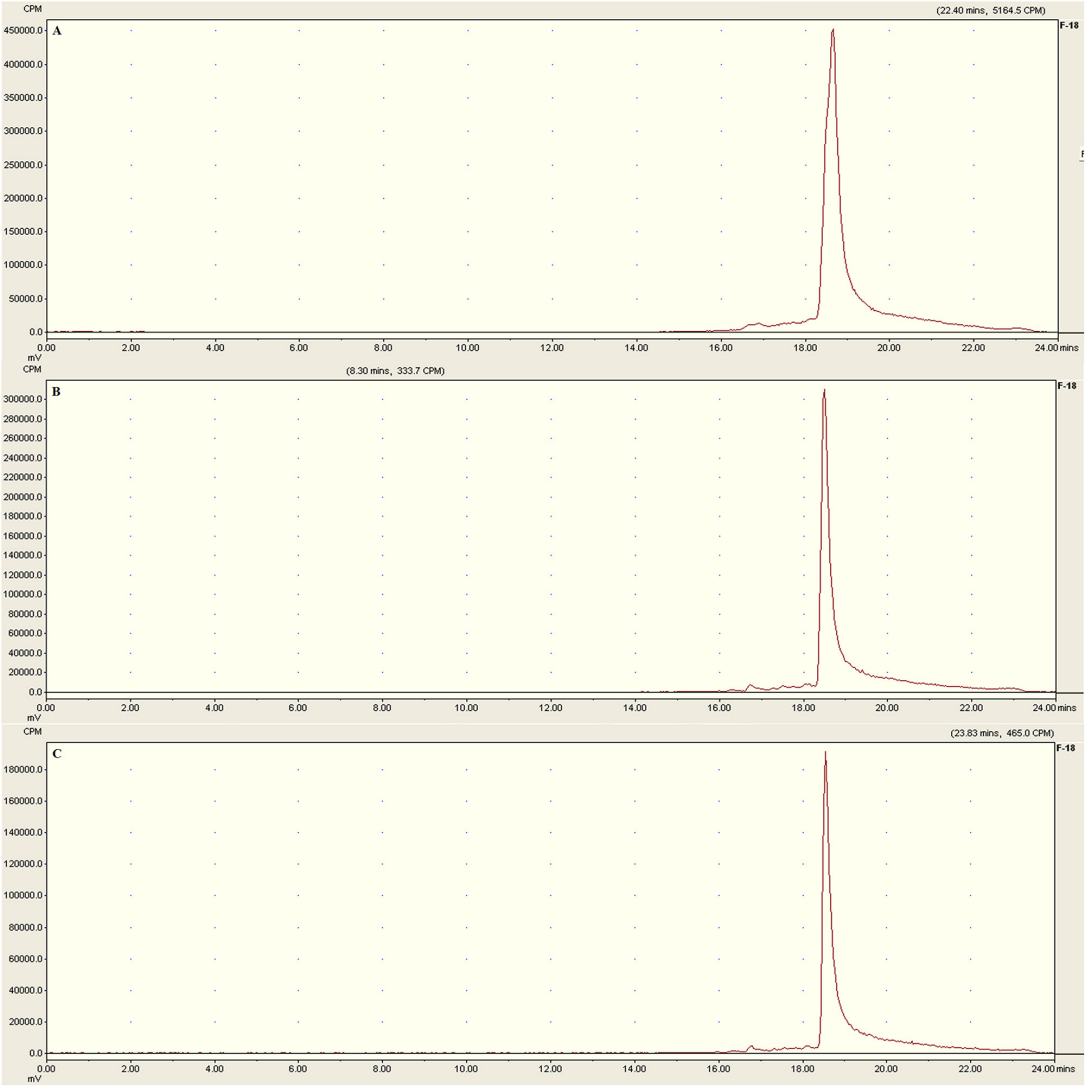
Analytical HPLC profile of [^18^F]AlF-NOTA-C6 after incubation in human serum at 37°C for (A) 0, (B) 2, and (C) 4 h.

The *in vitro* stability of [^18^F]AlF-NOTA-C6 was evaluated by radio-HPLC. As shown in Figs 4 and 5, after incubation in physiological saline at room temperature or in human serum at 37°C for 4 h, >95% of the radioactivity was observed in the form of [^18^F]AlF-NOTA-C6 (see S4 and S5 Figs). This analysis confirmed the absence of radioactive degradation products of [^18^F]AlF-NOTA-C6 at 2 and 4 h in physiological saline at room temperature or in human serum at 37°C.

### Cell uptake

**Fig 6:**
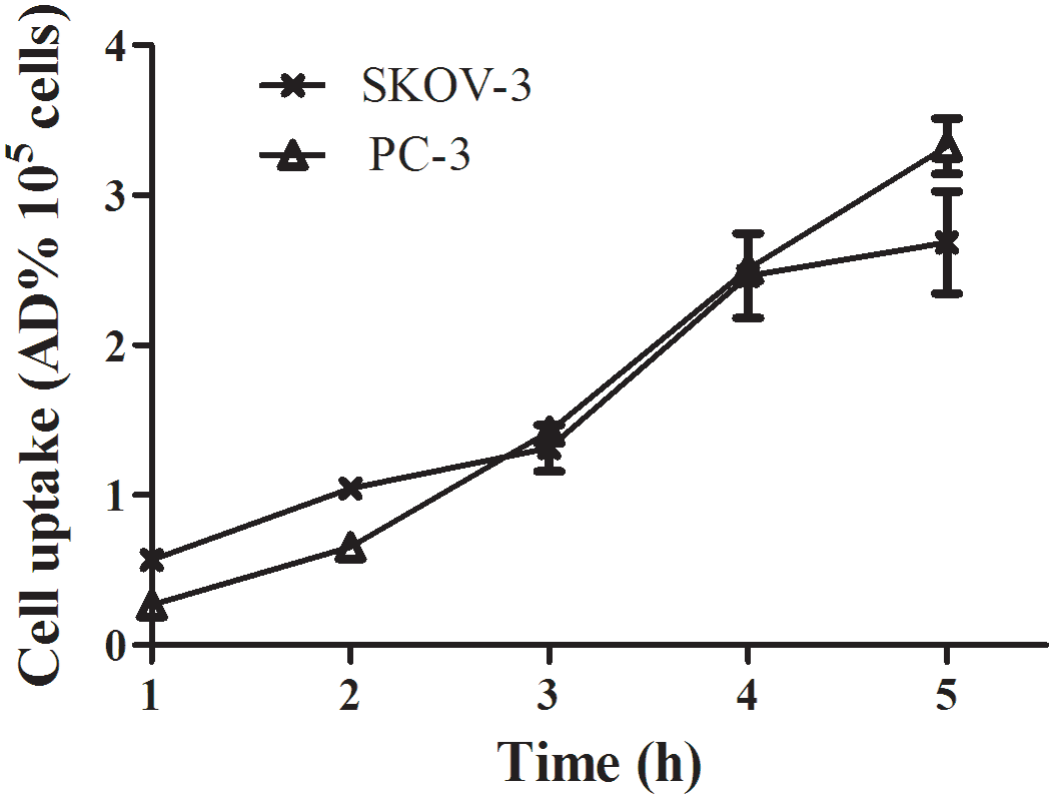
Cell uptake assays of [^18^F]AlF-NOTA-C6.

[^18^F]AlF-NOTA-C6 uptake in SKOV3 and PC3 cells increased over time in a linear fashion, as presented on Fig 6 (see S6 Fig). At 5 h of incubation, the uptakes of the added doses were 2.7%/10^5^ SKOV3 cells and 3.3%/10^5^ PC3 cells.

### Cell binding assay

**Fig 7:**
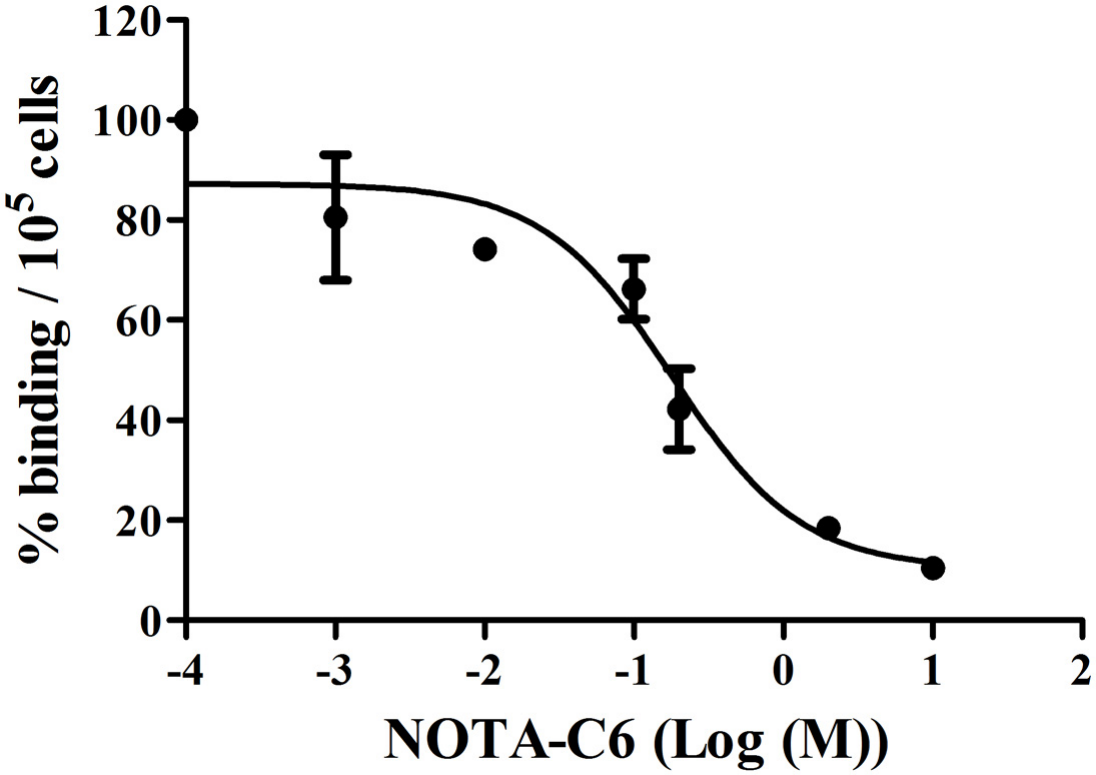
Competition of the binding of [^18^F]AlF-NOTA-C6 to SKOV3 cells with NOTA-C6.

The MMP2 binding affinity of [^18^F]AlF-NOTA-C6 was evaluated using NOTA-C6 as a competitive ligand. As shown in Fig 7, co-incubation with NOTA-C6 blocked the binding of [^18^F]AlF-NOTA-C6 to SKOV3 cells in a dose-dependent manner (see S7 Fig). The IC_50_ value of [^18^F]AlF-NOTA-C6 displacement with NOTA-C6 was 0.18 nM.

### *In vivo* PET imaging

To determine the feasibility of [^18^F]AlF-NOTA-C6 for noninvasive detection of solid tumors, PET imaging was performed on SKOV3 and PC3 tumor-bearing mice. For the SKOV3 tumor-bearing mice, the tumor radioactivity concentration of [^18^F]AlF-NOTA-C6 was visualized at 30 and 60 min PI; the uptake of the tumors was negligible at 120 min PI. Radioactive uptake was high in the kidney at different timepoints; compared with 30 min PI, radioactivity accumulation in the kidney at 60 and 120 min PI dropped, which indicates that the majority of the probe was cleared from the renal system.

**Fig 8:**
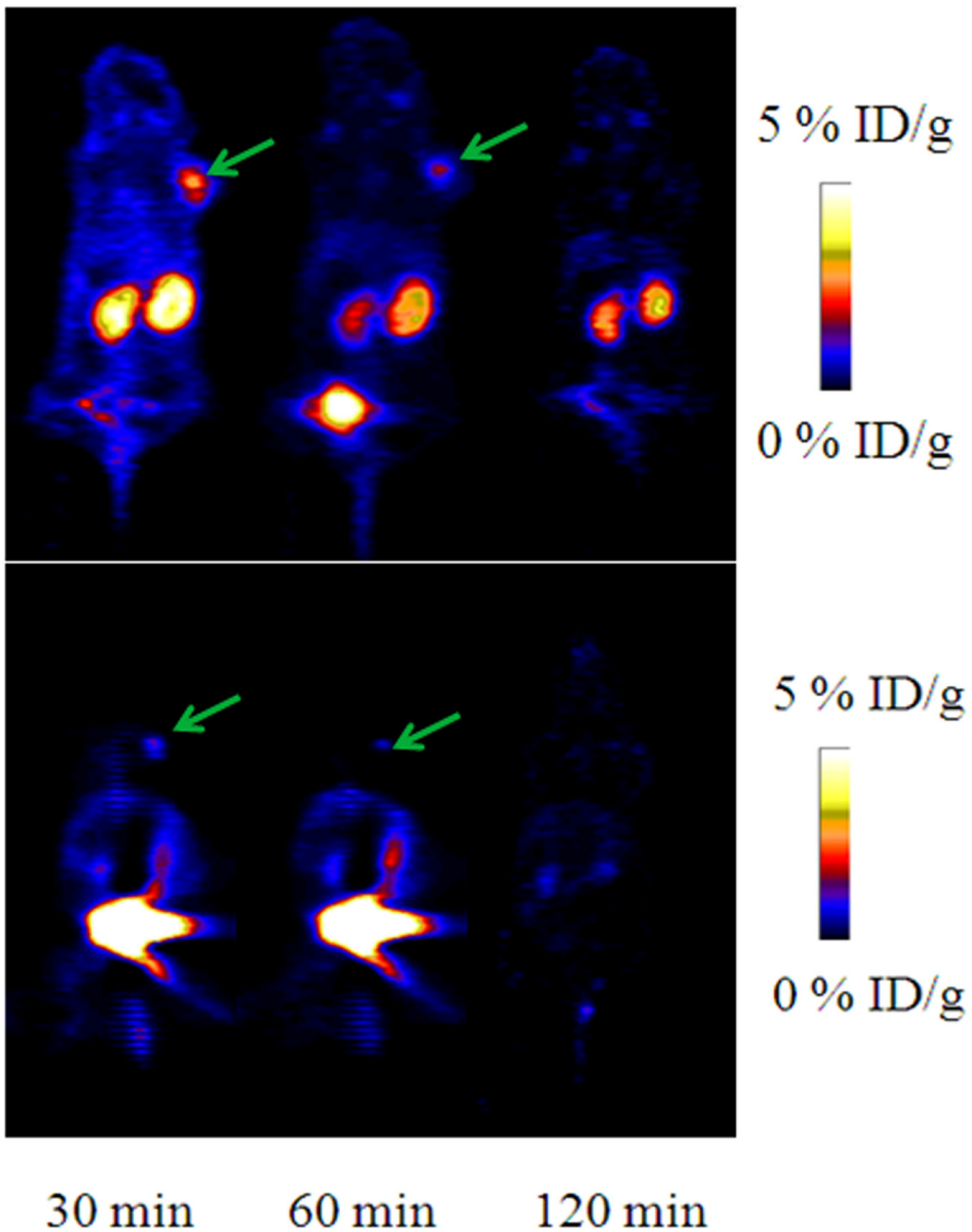
Representative microPET images of mice with SKOV3 tumors at 30, 60, and 120 min post-injection of [^18^F]AlF-NOTA-C6, with or without an overdose of C6 to pre-block (top row: non-blocked; bottom row: blocked). Arrows indicate the tumor sites.

**Fig 9:**
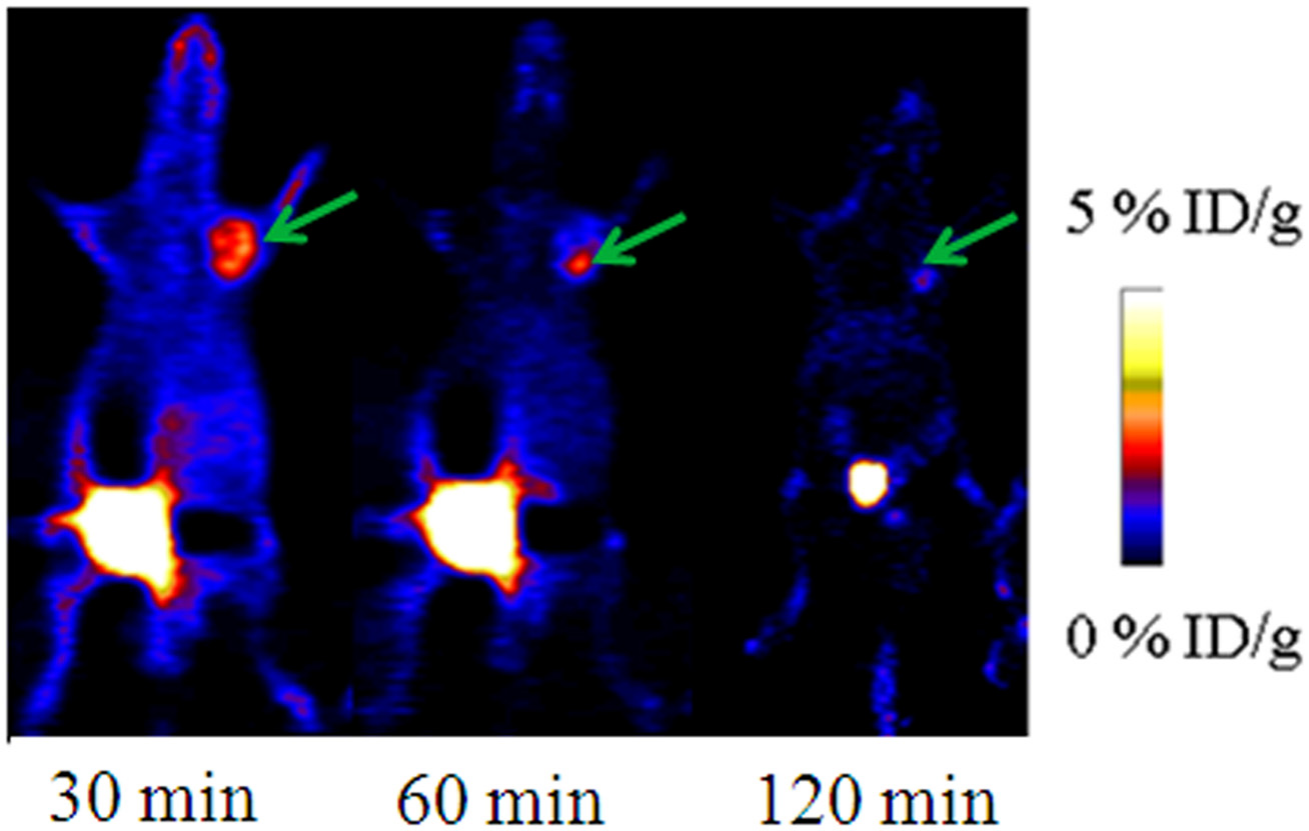
Representative microPET images of mice with PC3 tumors at 30, 60, and 120 min post-injection of [^18^F]AlF-NOTA-C6. Arrows indicate the tumor sites.

To confirm specificity, SKOV3 tumor-bearing mice were pre-blocked with an excess of C6 prior to the tracer injection. As seen in Fig 8, the pre-blocked mice had clearly lower activity in the same region compared with the non-blocked mice with tumors of similar size (see S8 Fig). For the PC3 tumor-bearing mice, the tumors were also detected via PET, and the greatest radioactivity concentration was identified at 30 min PI (Fig 9) (see S9 Fig).

### Biodistribution of [^18^F]AlF-NOTA-C6 in tumor-bearing mice

SKOV3 tumor-bearing mice were utilized for the biodistribution experiments (see S1 Table). At 30, 60, and 120 min PI, the tumor uptakes of [^18^F]AlF-NOTA-C6 were 1.20 ± 0.24% ID/g, 0.75 ± 0.25% ID/g, and 0.27 ± 0.14% ID/g, respectively. [^18^F]AlF-NOTA-C6 also showed great kidney accumulation with 7.14 ± 0.05% ID/g at 30 min PI, dropping to 4.72 ± 0.31% ID/g at 120 min PI. This finding indicated that the majority of the radioactivity was excreted via the kidneys into urine. Compared with the non-blocked animals, pre-injection of excess C6 caused a marked reduction in tumor tissue uptake (*P* < 0.05).

### Histopathology and MMP2 IHC of tumors

**Fig 10:**
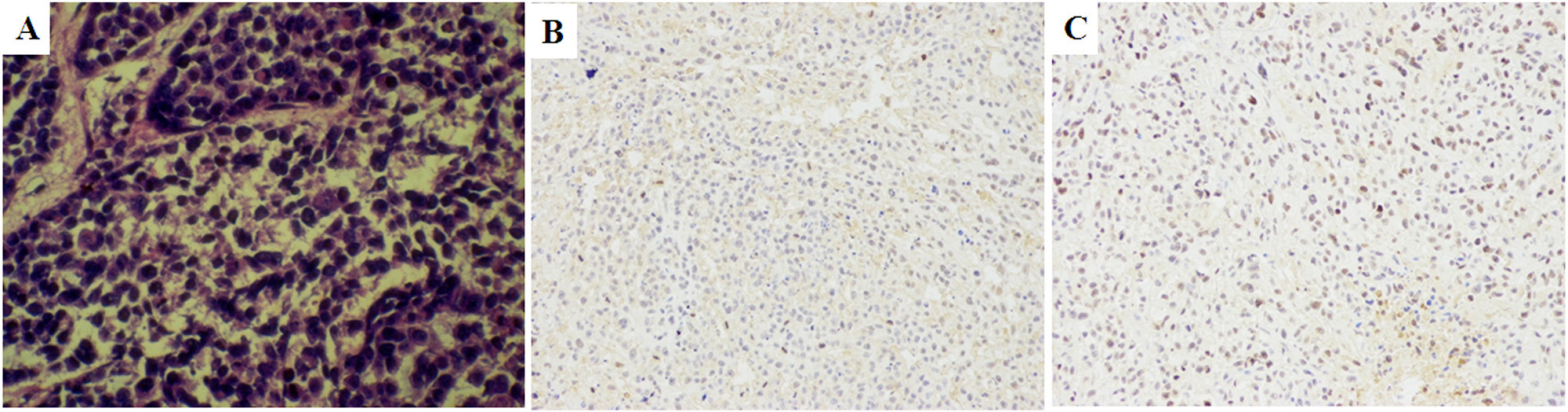
Representative photomicrographs of histological and immunohistochemical features. (A) H&E staining of an SKOV3 tumor 400 ×; (B) IHC staining of an SKOV3 tumor; (C) IHC staining of a PC3 tumor. The cells with brown granules in the cytoplasm were MMP2-positive.

The histological features of SKOV3 ovarian cancer was shown using H&E staining (see S10 Fig). The levels of MMP2 in the SKOV3 and PC3 cancer lesions were estimated by IHC staining, which showed high expression of MMP2 (+++ and ++, respectively; see S10 Fig). The results were explained in Fig 10.

## Discussion

MMP2 has been identified as a key MMP involved in tumor invasion, metastasis, and angiogenesis [28]. Overproduction and unrestrained activity of MMP2 has been linked to malignancy in a variety of tumors, including brain, prostate, colorectal, pancreatic, breast, and ovarian tumors, and has been associated with shorter disease-free survival [29–33]. As a result, MMP2 is a promising target for tumor imaging. In this study, the radiolabeling and feasibility of using an ^18^F-labeled peptide that targets MMP2 as a PET tracer for the imaging of MMP2 in tumors was explored.

The cyclic decapeptide C6 is a selective inhibitor of gelatinases [17]. [^18^F]-Fluoride is readily available via cyclotron production, can be obtained from commercial cyclotron facilities, and is the optimal PET radioisotope because of its short half-life. Complexing peptides with [^18^F]AlF is a simple procedure [19,23–25]. Thus, in this study [^18^F]AlF-NOTA-C6 was designed as a PET tracer for the imaging of MMP2 expression in tumors, and was conveniently prepared. The quality control assay suggested that solid-phase extraction is sufficient to remove unbound ^18^F without further HPLC purification. Analytical radio-HPLC indicated that the radiochemical yield was good and [^18^F]AlF-NOTA-C6 was stable *in vitro*.

For the present study, SKOV3 ovarian and PC3 prostate cell lines were selected for *in vitro* and *in vivo* experiments. These particular cell lines were chosen because previous studies have shown that SKOV3 and PC3 cells readily form subcutaneous tumors in rodents. Furthermore, MMP2 levels in SKOV3 and PC3 cell lines were significant [34,35], as confirmed by the IHC staining in this study.

The *in vitro* cell uptake results showed that [^18^F]AlF-NOTA-C6 was retained at favorable levels in SKOV3 and PC3 cells. Although Wang et al. [17] used a competitive cell-binding assay, an *in vitro* MMP2 inhibition assay, and an *in vitro* β-casein degradation assay to demonstrate that C6 binds to MMP2, in the present study, we performed a further competitive cell-binding assay on SKOV3 cells, using NOTA-C6 as a competitive ligand. When co-incubated with NOTA-C6, [^18^F]AlF-NOTA-C6 uptake in SKOV3 cells decreased in a dose-dependent manner. The cell uptake and competitive cell-binding assay results elucidated the binding of [^18^F]AlF-NOTA-C6 to MMP2.

In the PET imaging and biodistribution experiments, tumor radioactivity concentration was identified. Furthermore, when pre-blocked with unlabeled C6, [^18^F]AlF-NOTA-C6 uptake in tumors significantly decreased. These results also illustrated that [^18^F]AlF-NOTA-C6 targets tumors with high levels of MMP2. Although the blocking experiments with [^18^F]AlF-NOTA-C6 showed peptide binding to MMP2, minimal uptake of [^18^F]AlF-NOTA-C6 *in vivo* in the tumors was observed. It is presumed that the levels of free C6 peptide pre-injected into the mice were not sufficient. In addition, there was competition between [^18^F]AlF-NOTA-C6 and free C6; thus, complete blocking theoretically cannot be attained.

We also noted that in the *ex vivo* biodistribution, uptake was significantly blocked not only for tumors but also for other organs, especially blood, liver, and spleen. Previous reports have indicated that the mean serum level of MMP2 in cancer patients was significantly higher than in the control groups [36,37]. It is well known that the liver and spleen are organs rich in blood. Thus, we suggest that this characteristic may be why the radioactivity concentrations of these organs decreased after they were pre-blocked with unlabeled C6. Furthermore, even if there was some accumulation of MMP2 in the liver and spleen, because the ovaries and prostate are far from the liver and spleen, tumor detection in these two organs will not be influenced by higher uptakes in the liver and spleen.

Although C6 conjugated with Cy5.5 has been successfully used for the visualization of tumors *in vivo*, such as prostate PC3, glioma U87, and inflammation-induced colon tumors [17,18], to our knowledge, this study is the first time that C6 was labeled with [^18^F]-fluoride. Tumor imaging and biodistribution experiments have shown the potential of [^18^F]AlF-NOTA-C6 as an imaging agent for MMP2-positive tumors. ^125^I-, ^111^In-, and ^64^Cu-labeled CTT have previously been radiolabeled, and are particularly impressive tracers that target MMP2 [12–14]. CTT readily degraded *in vivo*. In contrast, C6 was more stable and had stronger gelatinase inhibitory activity than did CTT. Thus, we speculated that radiolabeled C6 may have some advantages over CTT. A comparison with ^125^I-labeled CTT indicated that [^18^F]AlF-NOTA-C6 reduced accumulation in the thyroid. Compared with ^111^In-DTPA-CTT, [^18^F]AlF-NOTA-C6 had higher uptakes in tumors and could display the tumors *in vivo*. However, compared with ^64^Cu-DOTA-CTT, it appears that the uptake ratios of tumor-to-blood of these two radiopharmaceuticals were similar (slightly >2-fold). The superiority of [^18^F]AlF-NOTA-C6 to ^64^Cu-DOTA-CTT was not indicated in our study. In addition, the absence of a detailed analysis of the correlation between MMP2 levels and the accumulation of [^18^F]AlF-NOTA-C6 in tumor tissue is another limitation of this study.

## Conclusion

[^18^F]AlF-NOTA-C6 was easy to synthesize and has good potential as an imaging agent that targets MMP2 in tumors.

## Supporting Information

S1 Fig The chemical structure of NOTA-C6. (TIF)

S2 Fig Schematic of [18F]AlF-NOTA-C6 radiosynthesis. (TIF)

S3 Fig Analytical HPLC profile of [18F]AlF-NOTA-C6. (TIF)

S4 Fig Analytical HPLC profile of [18F]AlF-NOTA-C6 after incubation in physiological saline at room temperature for (A) 0, (B) 2, and (C) 4 h. (TIF)

S5 Fig Analytical HPLC profile of [18F]AlF-NOTA-C6 after incubation in human serum at 37°C for (A) 0, (B) 2, and (C) 4 h. (TIF)

S6 Fig Cell uptake assays of [18F]AlF-NOTA-C6. (TIF)

S7 Fig Competition of the binding of [18F]AlF-NOTA-C6 to SKOV3 cells with NOTA-C6. (TIF)

S8 Fig Representative microPET images of mice with SKOV3 tumors at 30, 60, and 120 min post-injection of [18F]AlF-NOTA-C6, with or without an overdose of C6 to pre-block. (TIF)

S9 Fig Representative microPET images of mice with PC3 tumors at 30, 60, and 120 min post-injection of [18F]AlF-NOTA-C6. (TIF)

S10 Fig Representative photomicrographs of histological and immunohistochemical features. (TIF)

S1 Table Biodistribution of [18F]AlF-NOTA-C6 in SKOV3 tumor-bearing mice after injection with or without excess C6 to block receptors. (DOCX)
