# Supplementary figures and images for: Development of a Novel PET Tracer [18F]AlF-NOTA-C6 Targeting MMP2 for Tumor Imaging
Source: PLoS One. 2015 Nov 5;10(11):e0141668. doi: 10.1371/journal.pone.0141668 (PMC4634933; doi:10.1371/journal.pone.0141668)

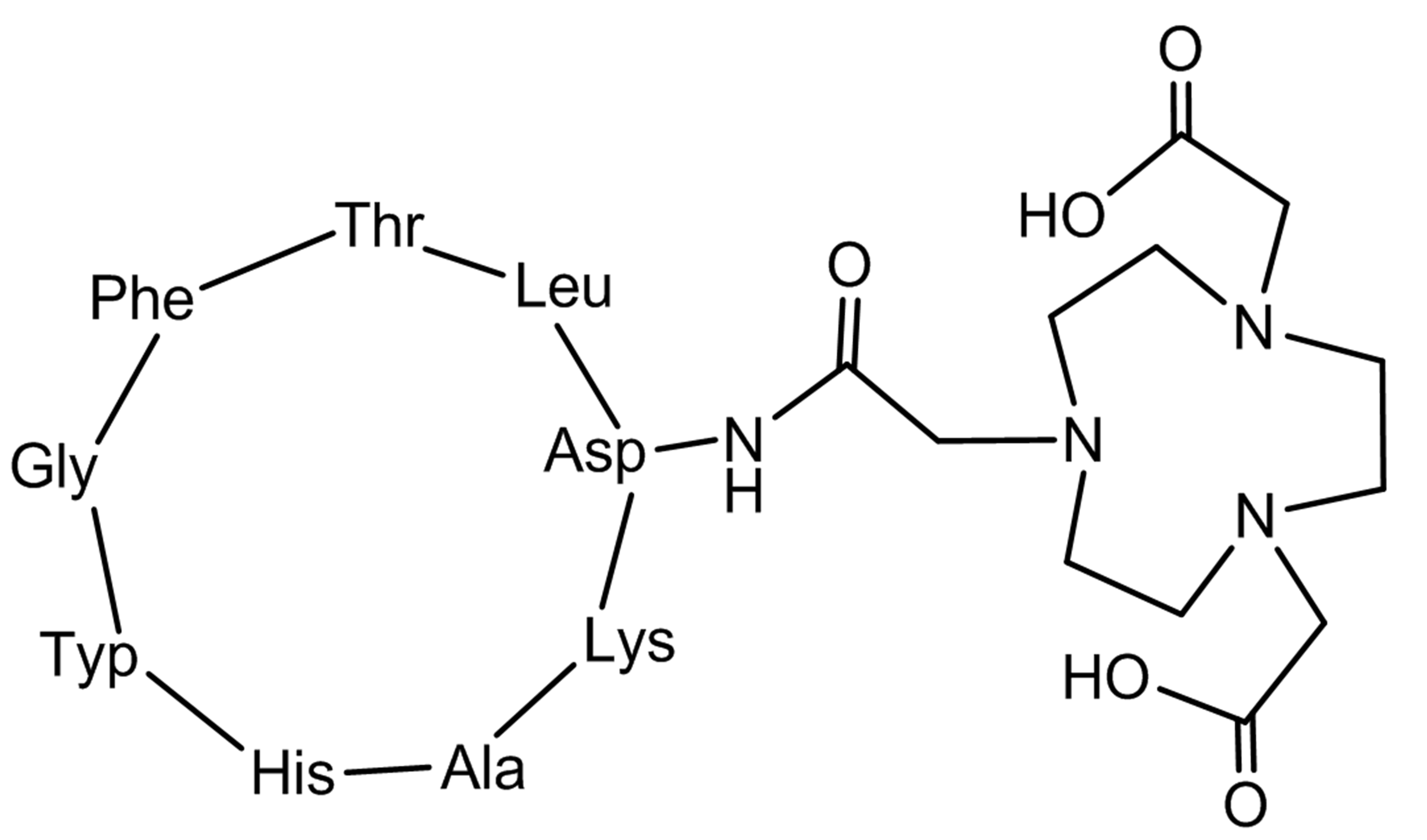

Supplement: S1 Fig — (TIF) [file pone.0141668.s001.tif]

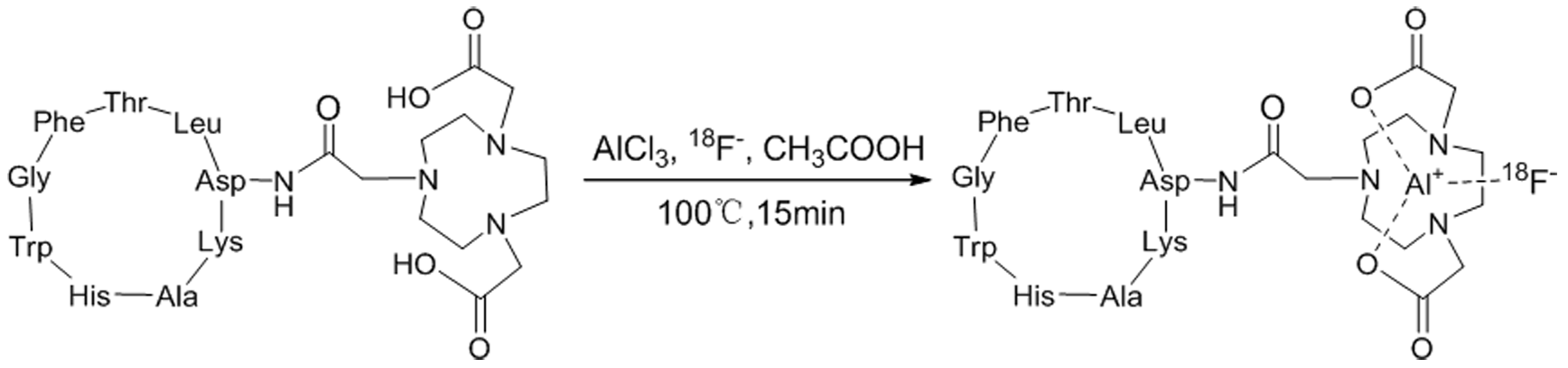

Supplement: S2 Fig — (TIF) [file pone.0141668.s002.tif]

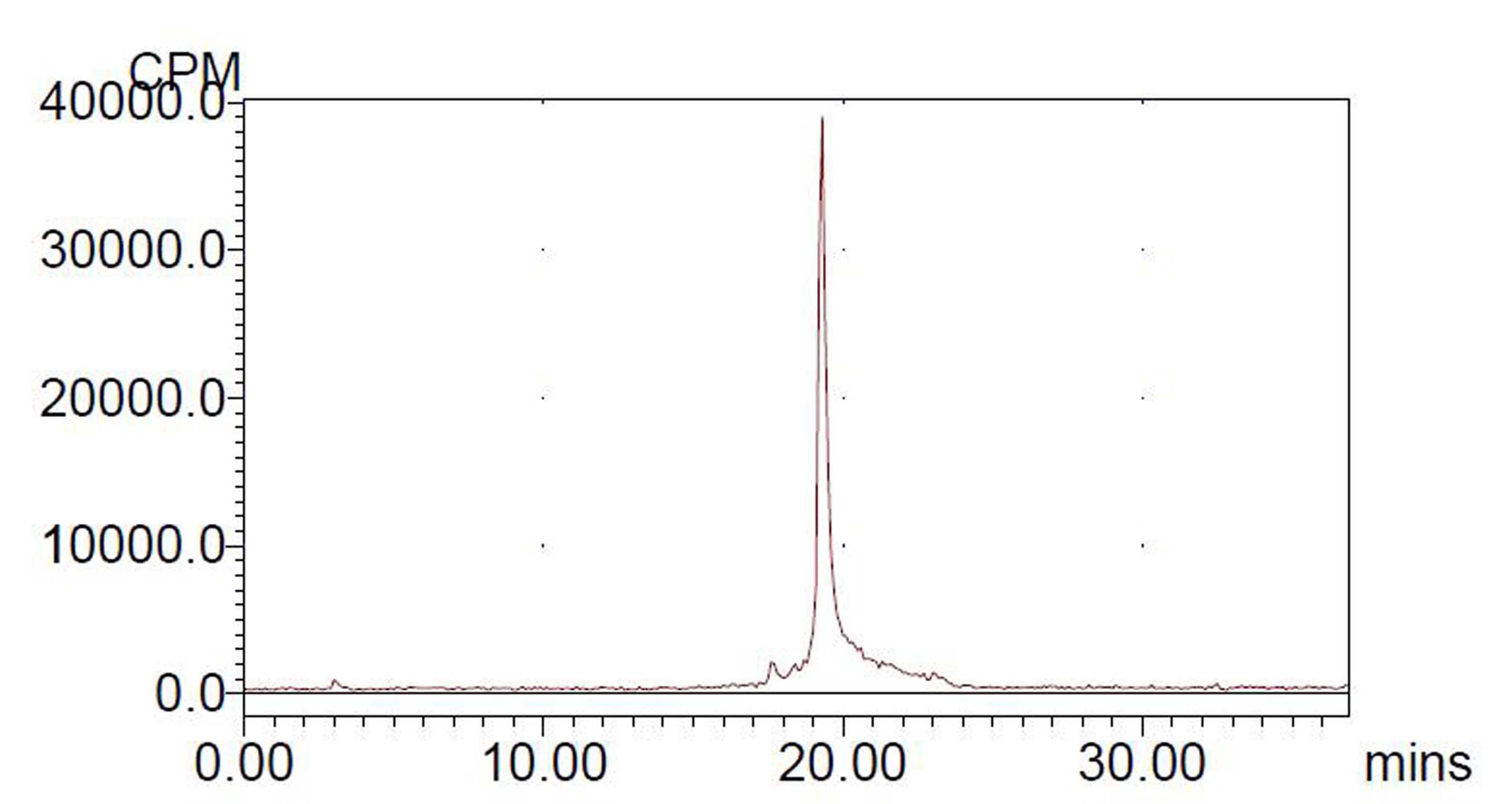

Supplement: S3 Fig — (TIF) [file pone.0141668.s003.tif]

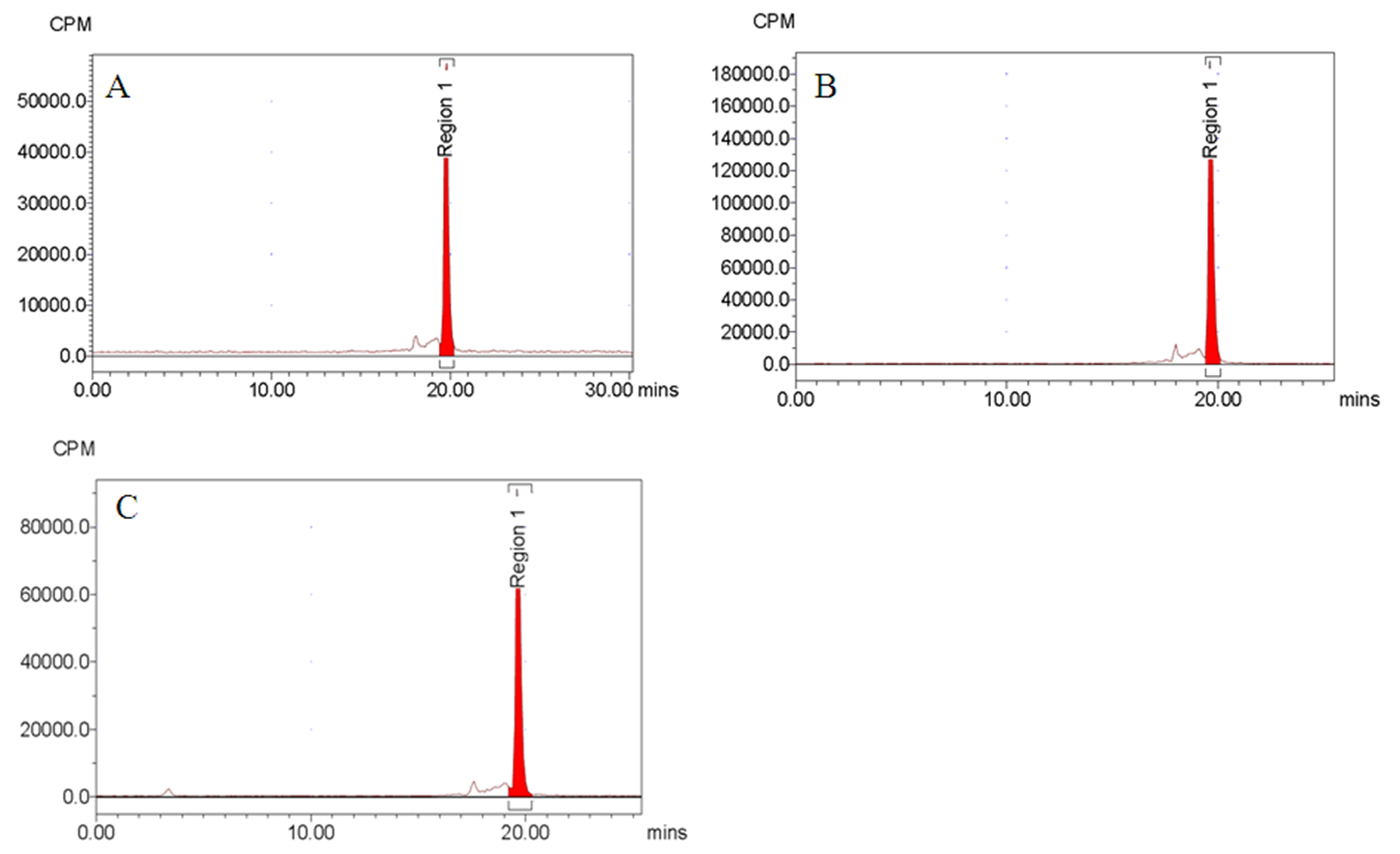

Supplement: S4 Fig — (TIF) [file pone.0141668.s004.tif]

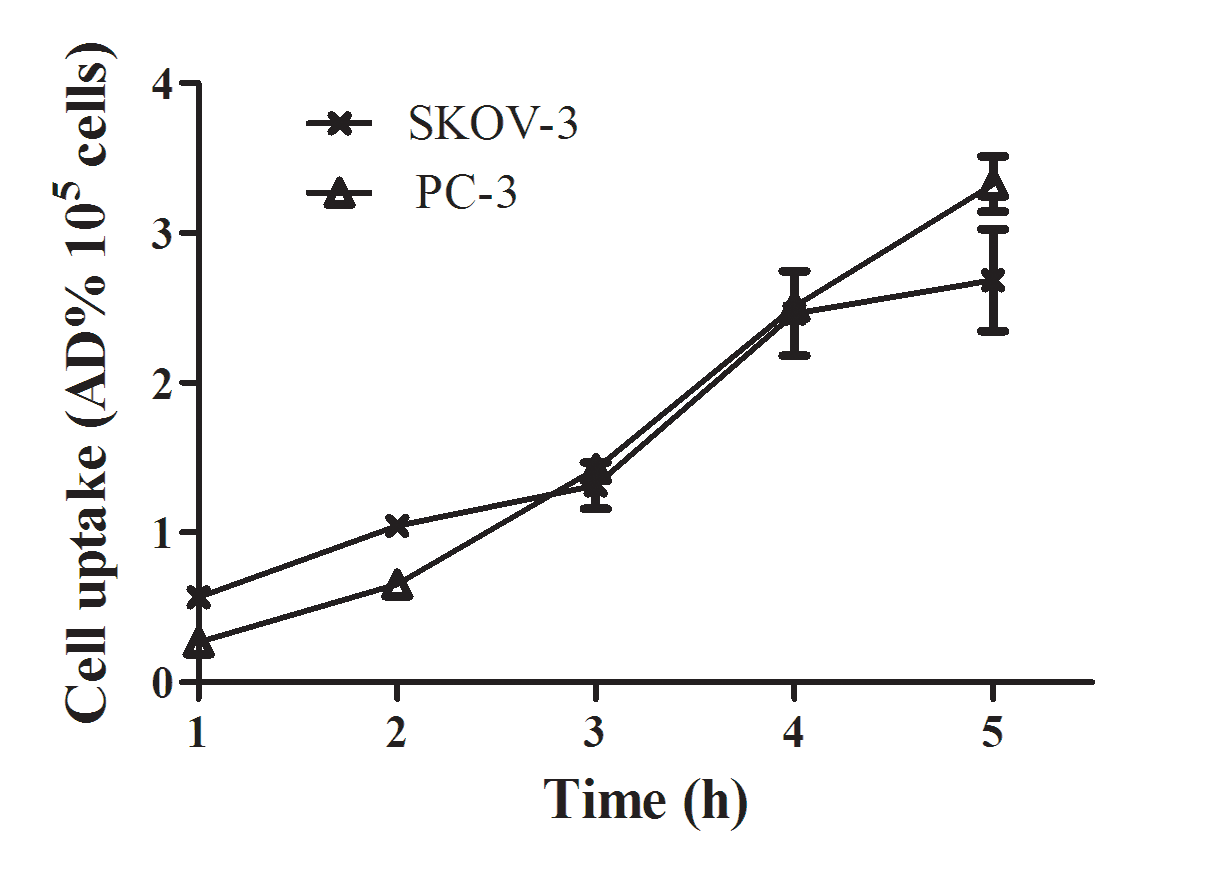

Supplement: S6 Fig — (TIF) [file pone.0141668.s006.tif]

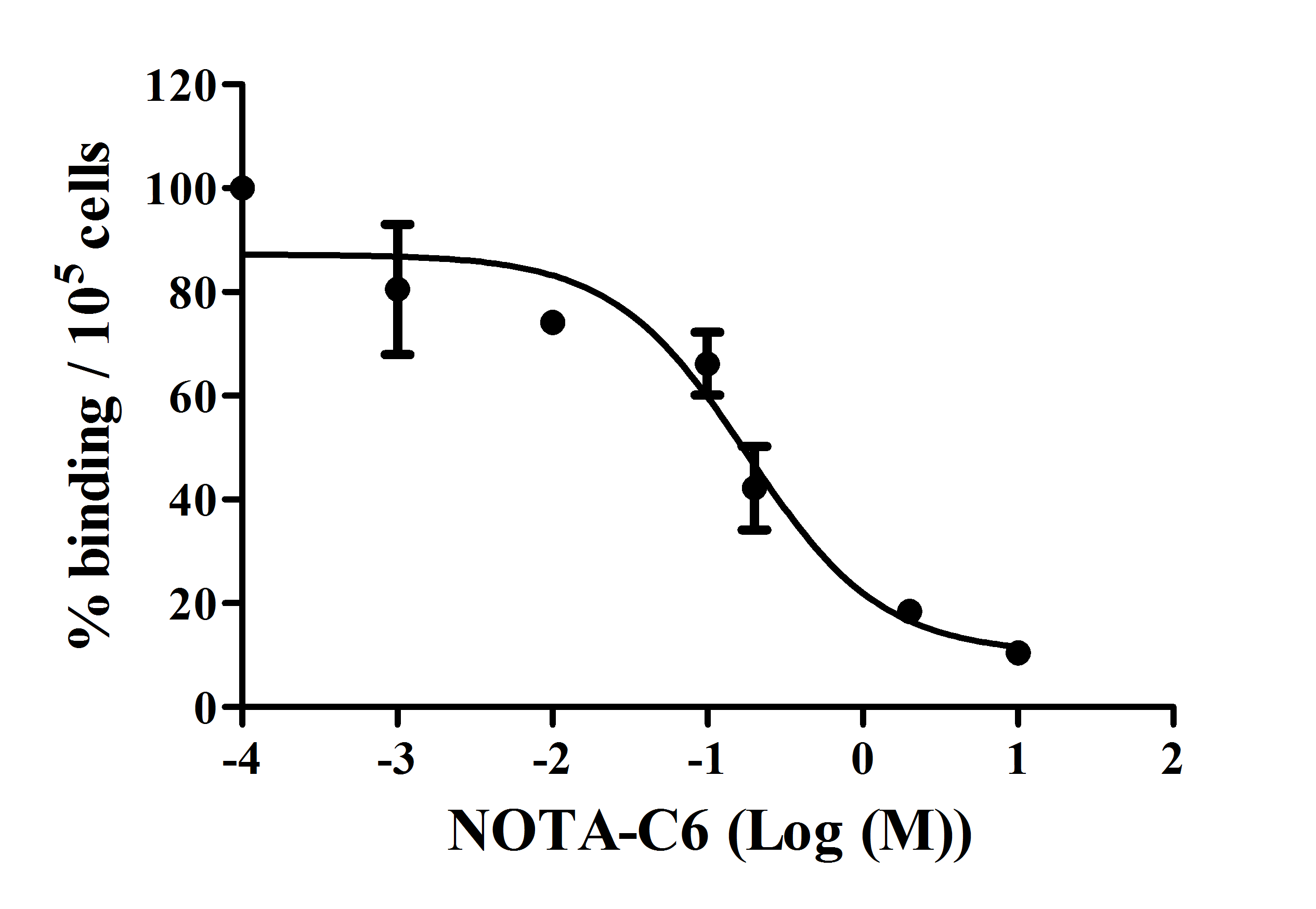

Supplement: S7 Fig — (TIF) [file pone.0141668.s007.tif]

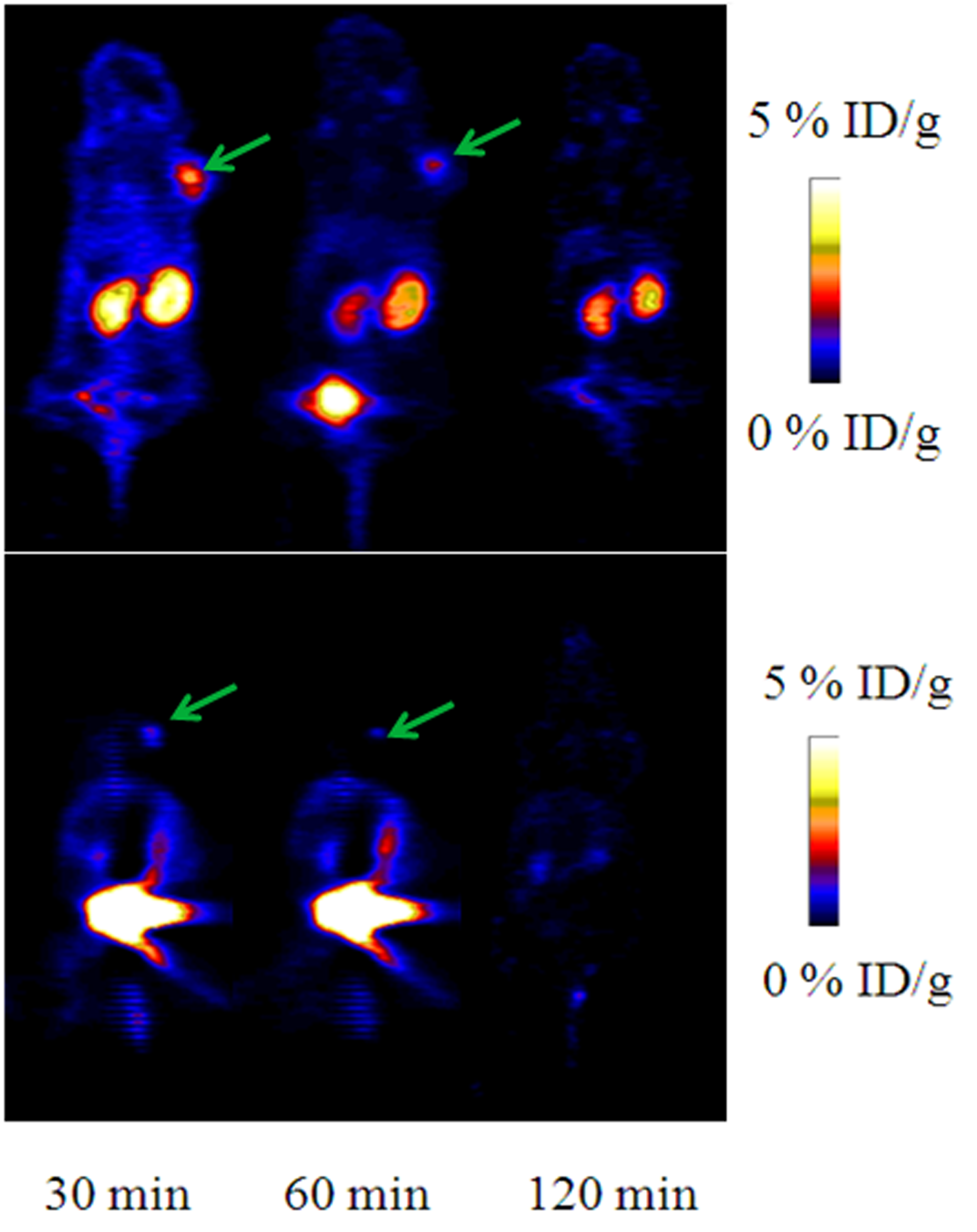

Supplement: S8 Fig — (TIF) [file pone.0141668.s008.tif]

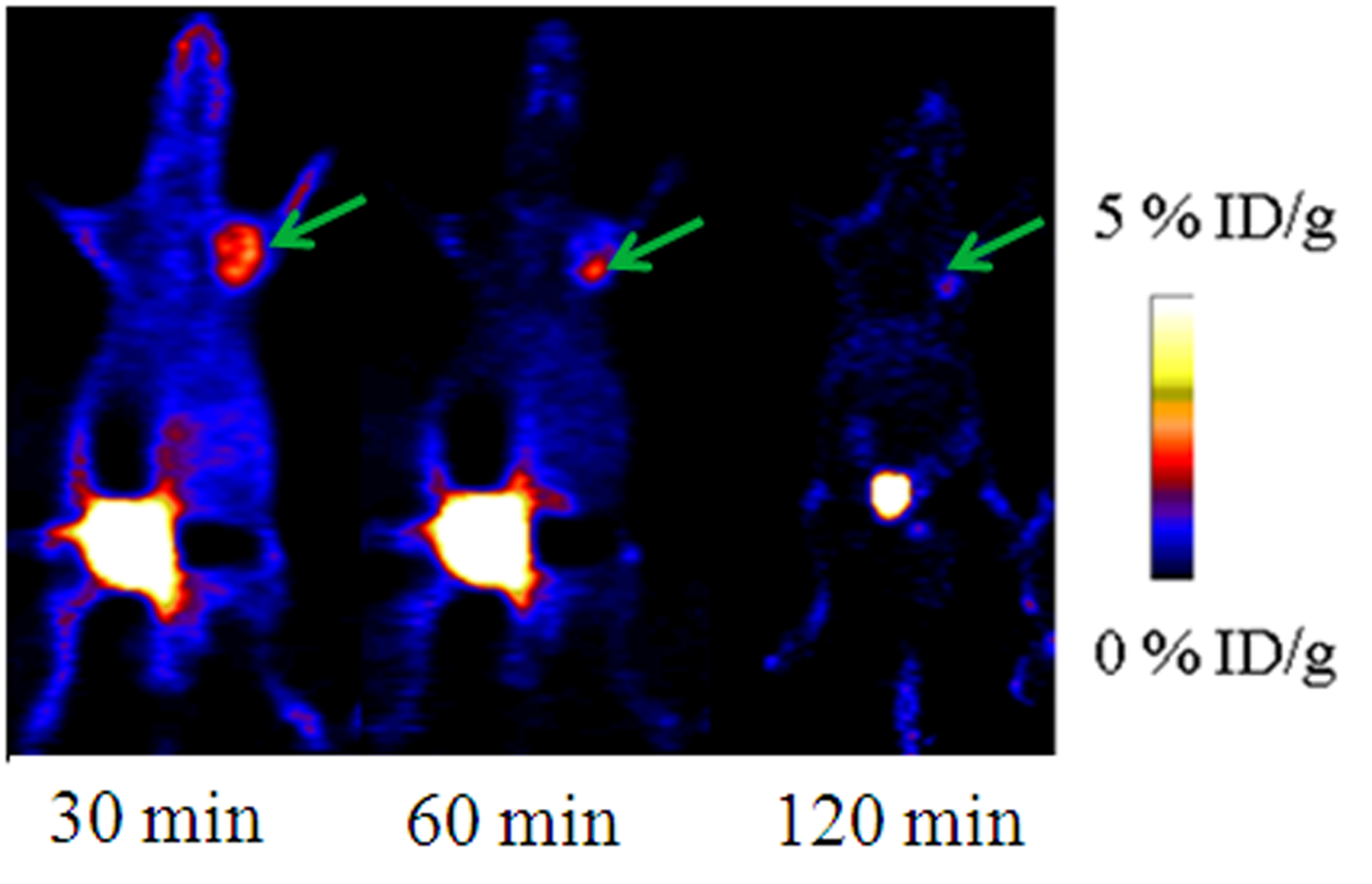

Supplement: S9 Fig — (TIF) [file pone.0141668.s009.tif]

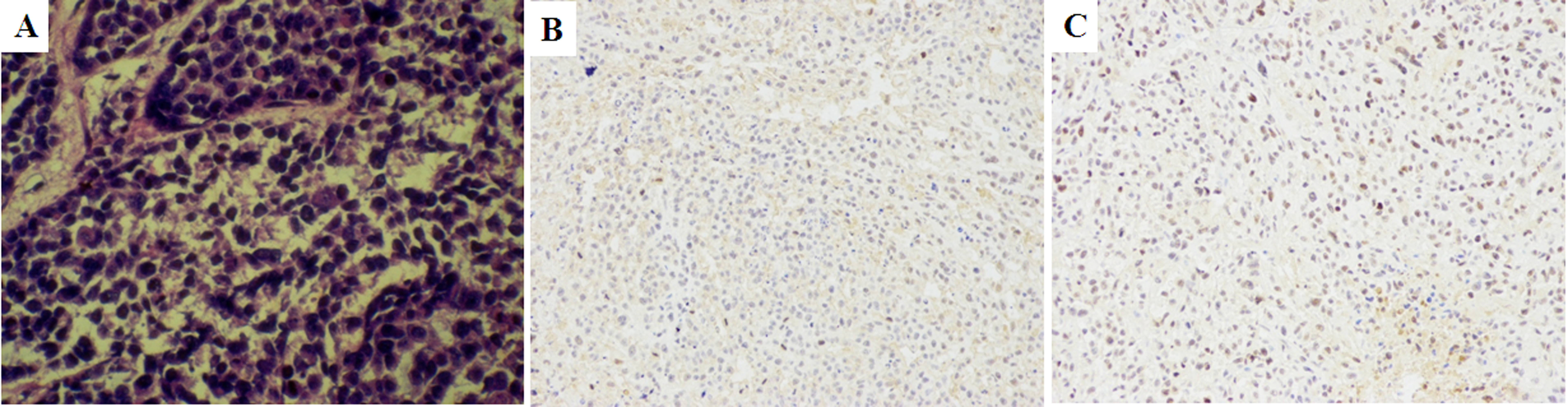

Supplement: S10 Fig — (TIF) [file pone.0141668.s010.tif]
