# Supplementary material for: Development of a Novel PET Tracer [18F]AlF-NOTA-C6 Targeting MMP2 for Tumor Imaging
Source: PLoS One. 2015 Nov 5;10(11):e0141668. doi: 10.1371/journal.pone.0141668 (PMC4634933; doi:10.1371/journal.pone.0141668)
Supplement: S1 Table — (DOCX) [file pone.0141668.s011.docx]

**Table 1.** Biodistribution of [^18^F]AlF-NOTA-C6 in SKOV3 tumor-bearing mice after injection with or without excess C6 to block receptors, % ID/g *

|  | Non-blocked | | | Blocked |
| --- | --- | --- | --- | --- |
|  | 30 min | 60 min | 120 min | 60 min |
| Blood | 1.84 ± 0.29 | 0.33 ± 0.10 | 0.35 ± 0.11 | 0.07 ± 0.01 |
| Brain | 0.08 ± 0.05 | 0.03 ± 0.02 | 0.03 ± 0.02 | 0.01 ± 0.00 |
| Heart | 0.46 ± 0.08 | 0.09 ± 0.03 | 0.11 ± 0.07 | 0.04 ± 0.01 |
| Liver | 1.79 ± 0.42 | 1.21 ± 0.53 | 1.20 ± 0.25 | 0.19 ± 0.02 |
| Spleen | 2.09 ± 1.02 | 1.69 ± 0.51 | 1.07 ± 0.29 | 0.06 ± 0.00 |
| Lung | 1.09 ± 0.16 | 0.31 ± 0.07 | 0.31 ± 0.03 | 0.10 ± 0.02 |
| Kidney | 7.14 ± 0.05 | 4.55 ± 1.38 | 4.72 ± 0.31 | 3.51 ± 0.32 |
| Stomach | 0.49 ± 0.31 | 0.21 ± 0.19 | 0.07 ± 0.02 | 0.05 ± 0.03 |
| Gut | 0.47 ± 0.09 | 0.13 ± 0.07 | 0.12 ± 0.07 | 0.04 ± 0.01 |
| Muscle | 0.57 ± 0.15 | 0.08 ± 0.04 | 0.21 ± 0.08 | 0.12 ± 0.02 |
| Pancreas | 0.35 ± 0.01 | 0.16 ± 0.14 | 0.09 ± 0.06 | 0.04 ± 0.02 |
| Sexual gland | 0.50 ± 0.12 | 0.05 ± 0.03 | 0.09 ± 0.02 | 0.05 ± 0.10 |
| Thyroid | 0.88 ± 0.13 | 0.16 ± 0.07 | 0.30 ± 0.16 | 0.17 ± 0.03 |
| Fat | 0.44 ± 0.08 | 0.18 ± 0.10 | 0.12 ± 0.07 | 0.04 ± 0.01 |
| Bone | 0.57 ± 0.04 | 0.41 ± 0.13 | 0.65 ± 0.07 | 0.32 ± 0.09 |
| Tumor | 1.20 ± 0.24 | 0.75 ± 0.25 | 0.27 ± 0.14 | 0.26 ± 0.14 |

*n = 4.
